# Body Image Concern and its Correlates among Male and Female Undergraduate Students at Assuit University in Egypt

**DOI:** 10.5539/gjhs.v6n5p105

**Published:** 2014-05-16

**Authors:** Walid El Ansari, Emily Dibba, Shokria Labeeb, Christiane Stock

**Affiliations:** 1 Faculty of Applied Sciences, University of Gloucestershire, Gloucester, United Kingdom; 2 Unit for Health Promotion Research, Institute of Public Health, University of Southern Denmark, Denmark; 3 Faculty of Nursing, Assiut University, Assiut, Egypt

**Keywords:** Egypt, body image concern, gender, student health, depressive symptoms

## Abstract

**Introduction:**

This cross-sectional study examined variables associated with body image concern (BIC) and whether these associations differed between female and male students in Egypt. During the period 2009-2010, 3271 undergraduate students (1663 females, 1504 males) at Assuit University in Egypt completed a self-administered questionnaire that assessed BIC and other socio-demographic and health related variables.

**Methods:**

Based on Cooper et al.’s Body Shape Questionnaire the authors categorized BIC into ‘no BIC’; ‘mild BIC’; and ‘moderate/marked BIC’. Multifactorial linear regression analysis examined the association between BIC and BMI, body image perception, lifestyle (physical activity, nutrition, smoking) and mental well-being variables (quality of life, finances-related stress, perceived stress, perceived health, depressive symptoms).

**Results:**

About 40% of the female students and 25.6% of male students reported having mild to marked BIC. The correlates of BIC did not exhibit striking differences between male and female students. For both genders, BIC was positively associated with BMI, body image perception as being too fat and with depressive symptoms. Self-rated health was inversely associated with BIC.

**Conclusion:**

These findings suggest that health promoting strategies should address the co-occurrence of depressive symptoms and BIC, and should furthermore pay due attention to higher prevalence of BIC among female students.

## 1. Introduction

Body image can be defined as a person’s perception, feelings and thoughts about his or her body, usually conceptualized as incorporating body size estimation, the evaluation of body attractiveness, as well as various emotions associated with body shape and size (Grogan, 2006). BIC is thus a multi-dimensional construct, frequently defined as the degree of satisfaction about oneself in terms of size, shape and general appearance (Cash & Deagle, 1997).

Traditionally, in the Eastern Mediterranean Region (EMR) and Arab societies, plumpness was considered as female beauty and femininity. However, recent EMR studies revealed increasingly westernized views of the ideal body image (Yahia, El-Ghazale, Achkar, & Rizk, 2011), where thin body image is now considered a beautiful feature of females (Musaiger, Bin Zaal, & D’Souza, 2012). This adaption to the westernized culture may increase the risk of having body image concern (BIC). Such effects can be illustrated by findings in the United Arabic Emirates, where the prevalence of disordered eating attitudes has risen (Thomas, Khan, & Abdulrahman, 2010). Thomas et al. (2010) concluded that such prevalence of disordered eating attitudes may in part arise from body image dissatisfaction and ultra-thin body image ideal.

Young people might be especially vulnerable to pressure to follow perceived standards of physical appearance, during an age that is important for the development of one’s identity in relation to physical self-evaluation and self-worth (Arnett, 2000). University students’ social environment particularly stimulates an increased awareness of social norms connected to appearance and attractiveness that escalates their risk for employing unhealthy body-change approaches (Bergstrom, Neighbors, & Lewis, 2004). BIC is linked to unhealthy outcomes such as unnecessary dieting, excessive weight control, low self-esteem and self-worth, and with serious life and economical costs such as in eating disorders (Smink, van Hoeken, & Hoek, 2012). Additionally, BIC is associated with a complex range of variables. These include lifestyle characteristics such as physical activity (PA) (Johnson, Fallon, Harris, & Burton, 2013), nutrition behaviors (Bener & Tewfik, 2006), smoking (Kendzor, Adams, Stewart, Baillie, & Copeland, 2009), mental well-being variables [e.g., quality of life (QoL), perceived stress, perceived health, depressive symptoms], body image perception (El Ansari, Clausen, Mabhala, & Stock, 2010a; Kjaerbye-Thygesen, Munk, Ottesen, & Kruger Kjaer, 2004), and body mass index (BMI) (Garrusi, Garousi, & Baneshi, 2013).

The literature suggested several gaps in current knowledge. First, studies examined BIC in the West (Rodgers, Ganchou, Franko, & Chabrol, 2012), while less research scrutinized BIC in EMR countries (Korn, Gonen, Shaked, & Golan, 2013; Musaiger & Al-Mannai, 2014; Yahia et al., 2011; Zeeni, Gharibeh, & Katsounari, 2013). Secondly, EMR studies that assessed body image focused on either eating disorders, self image or dieting to conclude that further BIC research was required (Korn et al., 2013; Yahia et al., 2011; Zeeni et al., 2013). Thirdly, few studies (Musaiger & Al-Mannai, 2014) evaluated the actual BIC among EMR university students. The sparse research that actually assessed BIC seems deficient, as studies examined only female university students, despite that men similarly experience altered perceptions of their bodies (Rodgers et al., 2012). For instance, in the UAE, Schulte and Thomas (2013) concluded that eating disorders prevention strategies should address the needs of females and males and consider potential depressive co-morbidity. Fourthly, BIC has been scrutinized in relation to the role of the media (magazines, television), whereas less research focused on the association of BIC with BMI, and with many lifestyle features and mental well-being variables.

The current study contributes to the existing knowledge and evidence base by examining the association between self-reported BIC of male and female university undergraduate students in Egypt and BMI, lifestyle aspects, and mental well-being variables. The three specific objectives were to:

(a) Describe the prevalence of three levels of BIC and potentially associated variables by gender.
(b) Assess the associations of BIC with a range of variables, e.g., BMI, body image perception, lifestyle characteristics, and mental well-being (bivariate associations) by gender.
(c) Assess the correlates of BIC for males and females, and examine whether these BIC correlates differ by gender (multifactorial analysis).

## 2. Materials and Methods

### 2.1 Data Collection Procedures

The data evaluated was collected as part of the General Student Health Survey in Assuit University, Egypt (El Ansari, Labeeb, Moseley, Kotb, & El-Houfy, 2013a; El Ansari et al., 2012a). This university was selected due to its research interests, existing contacts, and successful previous collaboration. The university ethics committee provided ethical approval for the study. Students attending lectures of randomly selected courses were provided with self-administered questionnaires towards the end of a lecture (or during allocated time) which were then collected after completion. To ensure that the students could participate on a voluntary basis, students were informed that their participation was both voluntary and anonymous (no incentives were provided), each questionnaire had an information sheet outlining the research objectives, and students who remained in class to complete the survey consented to their participation. A convenient sample of students was sought at all participating faculties (≈ 10% of students at each Faculty). Data were confidential and protected at all stages of the study. All data were computer-entered by a single person to minimize data entry errors and to maximize quality.

### 2.2 Sample

The present study comprised 3271 undergraduate students. A total of 104 students with gender not recorded were excluded from the analysis, leaving 1663 females (52.5%, M age 18.6, SD 1.2) and 1504 males (46.5%, M age 19.3, SD 1.6). Data were collected (academic year 2009-2010) at eleven faculties of Assiut university, Egypt to include Faculties of Physical Education (n = 168, M age 19.6, SD 0.8), Business (n = 604, M age 18.9 years, SD 1.3), Engineering (n = 572, M age 20.2, SD 1.5), Education (n = 461, M age 18.7, SD 0.9), Art (n = 424, M 18.8 years, SD 1.4), Social Work (n = 328, M age 18.6, SD 1.3), Sciences (n = 206, M age 18.0, SD 0.8), Computers and Information (n = 137, M age 17.5, SD 0.7), Veterinary Medicine (n = 131, M age 17.8, SD 1.4), Specific Education (n = 119, M age 18.5, SD 0.8), and Agriculture (n = 50, M age 17.9, SD 0.9). Based on the number of returned questionnaires, the response rates were approximately 90%.

### 2.3 Students’ Health Questionnaire

The study was a general student health survey implemented in several countries (El Ansari & Stock, 2010c; El Ansari et al., 2007, 2010a, 2011a and b, 2012a and b, 2013a, b, c and d, 2014). The original questionnaire was in English, and it was translated into Arabic using two independent forward translations. An expert committee (three members of staff from Assiut University) also reviewed the translated questionnaire to ensure face validity. In addition, the research team’s native Arabic speakers (WEA and SL) examined the translation quality, scrutinized the content of the translated questionnaire, reviewed any instances of disagreement, misunderstandings, mistranslations or inaccuracies, and made decisions accordingly (El Fakir et al., 2014). The translations focused on and ensured the equivalence of meaning, and the assessment of the same attributes, i.e., cross-cultural and cross-language equivalence (Beaton, Bombardier, Guillemin, & Ferraz, 2000). The final translated Arabic questionnaire was piloted, with very little comments returned that were amended.

The survey provided information on BIC, socio-demographic factors (gender, age), BMI, lifestyle characteristics [e.g., physical activity (PA), nutrition, smoking], mental well-being (e.g., QoL, finance-related stress, perceived stress, perceived health, depressive symptoms), and body image perception.

*Body Image Concern (BIC) (8 items)* was assessed by the Body Shape Questionnaire (Cooper, Taylor, Cooper, & Fairburn, 1987; Evans & Dolan, 1993) measuring levels of concern with shape in the last 4 weeks (a six-point Likert scale, 1 = “Never” to 6 = “Always”). Sample items include: “Have you been so worried about your shape that you have been feeling you ought to diet”; “Have you noticed the shape of others and felt that your own shape compared unfavorable”; and “Have you not gone out to social occasions because you have felt bad about your shape?”. The overall score ranged from 8-48 points. Cronbach’s alpha for the scale was 0.73. For analysis, we categorized BIC into four levels: (a) no BIC (8-18 points); (b) ‘mild’ BIC (19-25 points); (c) ‘moderate’ BIC (26-33 points); or (d) ‘marked’ BIC (34-48 points), based on Taylor (1987). Due to insufficient numbers, the ‘moderate’ and ‘marked’ BIC categories were collapsed into a single category entitled ‘moderate/marked’ BIC (26-48 points), thus finally ending up with three BIC categories.

*Body mass index (BMI) (2 items)* was calculated from measured weight (kilogram (kg)) and height (meter (m)) using metric BMI Formula (BMI = (kg/m^2^)). Measurements were undertaken using Seca Digital Weight and Height Scale, with height measured to nearest 0.1 centimeter (barefooted), and body weight measured to the nearest 0.1 kg (light clothing, no footwear) (El Ansari, El Ashker, & Moseley, 2010b). As per WHO guidelines (WHO, 2000), we categorized BMI into underweight (BMI < 18.5kg/m^2^), normal (18.5 ≤ BMI ≤ 24.9 kg/m^2^), overweight (BMI ≥ 25.0 kg/m^2^).

*Physical Activity (PA) (2 items)* included the following questions: “On how many of the past 7 days did you participate in moderate exercise for ≥ 30 minutes?”, and “On how many of the past 7 days did you participate in vigorous exercise for ≥ 20 minutes?”. For both questions, participants answered with a rating from 1-7 days. We used the cut-offs of < 5 days/week (moderate exercise) and < 3 days/week (vigorous exercise) in the current analysis in line with the American Heart Association guidelines (Haskell et al., 2007). We computed two PA categories: ‘Achieving PA guidelines’ (participate in ≥ 5 days of moderate exercise, for ≥ 30 minutes/day; or participate in ≥ 3 days of vigorous exercise for ≥ 20 minutes/day) versus ‘Not achieving PA guidelines’.

For *Nutrition (4 items)* the students completed a semi-quantitative food frequency questionnaire (4 indicator variables) that measured their consumption of sweets, cakes/cookies, snacks and fast/canned food as well as other food groups that can be considered important when assessing dietary habits (Mikolajczyk, El Ansari, & Maxwell, 2009; El Ansari et al., 2012a). The introductory question “How often do you eat the following foods?” asked participants about the frequency of their usual consumption of each food group separately. A 5-point scale was used and included the following response options: several times a day (5 points); daily; several times a week; 1–4 times a month; and never (1 point)]. Using these points, all food items were employed to construct a total score labeled: ‘High calorie diet score’ (4 - 20 points). Cronbach´s alpha for the scale was 0.65. For the current analysis, we categorized this High calorie diet score into: ‘low’ (1st tertile), ‘medium’ (2nd tertile), and ‘high’ (3rd tertile). No formal test of validity was performed, although the questionnaire was very similar to other validated food frequency questionnaires (Osler & Heitmann, 1996).

*Smoking* was assessed by the following question (*1 item*): “Within the last three month, how often did you smoke?” (3 point scale rating: “Daily”, “Occasionally”, “Never”, which were then re-coded into two binary variables (“Daily”, “Non/Occasionally”).

*Quality of Life* *(QoL) (1 item)* was assessed by QoL measurement charts, based on COOP/WONCA charts (Bruusgaard, Nessøy, Rutle, Furuseth, & Natvig, 1993). “If you consider the quality of your life: How did things go for you in the last four weeks?” [“Very badly” (5 points); “Badly” (4 points); “Intermediate” (3 points); “Quite well” (2 points); or “Very well” (1 point)]. We re-coded these five options into two categories: “Lower” (“very badly”, “badly” or “intermediate”); and “Higher” (“quite well” or “very well”).

*Finance-related stress* *(1 item)* included the following question: “To what extent do you feel burdened in the following area(s)?: “Finance” (rated using a six-point Likert scale: “Not at all” (1 point), “Very strongly” (6 points). For the current analysis, we created a binary variable by combining the three lower categories into one category “lower”, and combining the three higher categories to “higher”.

*Perceived stress* *(4 items)* was measured with the 4-item Perceived Stress Scale (PSS-4) (Cohen, Kamarck, & Mermelstein, 1983) (five-point Likert scale for each item). A total PSS score was calculated in line with Cohen et al. (1983), and then recoded for the current analysis using median split into two categories: “low” (≤ median); and “high” (> median).

*Self-rated health* *(1 item)* included the following question: “How would you rate your health in general?” rated on a five-point scale response (“Excellent”, “Very good”, “Good”, “Fair”, and “Poor”) (ACHA, 2006; Potthoff, Schroeder, Reis, & Klamert, 1999). For the current analysis we re-coded it into two categories: ‘Higher’ perceived health (“Excellent” and “Very good”); and ‘Lower’ perceived health (“Good”, “Fair”, and “Poor”).

*Depressive symptoms (19 items)* was measured using a modification of the Beck Depression Inventory (M-BDI) (Beck, Steer, Ball, & Ranieri, 1996). The modification of the original BDI included two approaches: (a) the four items per symptom which assessed the specific symptom’s intensity in the original BDI were replaced by a single statement per symptom with a six point Likert scale measuring its frequency in the last 4 weeks (with the two extreme categories labeled as 1 = ‘Never’, 5 = ‘Almost Always’), (b) One item (“I lost interest in sex”) was not included in the current survey as it was deemed as culturally inappropriate in a country of predominately the Muslim faith. The reduction in the number of items per symptom is consistent with other recent modification of BDI (BDI-II). The authors of the M-BDI have demonstrated its construct validity and measurement equivalence as compared to the original BDI (Schmitt et al., 2006). We calculated the sum of the 19 items of the M-BDI (7-35 points) and grouped depressive symptoms for the current analysis into two binary categories: Low (≤ 4^th^ quintile); and High (>5^th^ quintile) depressive symptoms. Cronbach’s alpha for the scale was 0.87.

The *body image perception (1 item)* question was adapted from the health behavior in school-aged children study (Currie, Sandal, Boyce, & Smith, 2001). The introductory question, “In your opinion are you…” asked participants about the perception of one’s body image (5-point scale: “Far too thin”, “A little too thin”, “Just right”, “A little overweight”, or “Very overweight”). For the current analysis, were re-coded the five options into three variables (‘Too thin’, ‘Just right’, ‘Too fat’).

### 2.4 Statistical Analysis

SPSS 20.0 (IBM Corp. Armonk, NY) was employed to compute frequencies and proportions and to conduct the statistical analysis. For several variables, some of the response options were combined to satisfy the assumption of adequate cell size for analysis.

For the first objective, frequencies were reported by three levels of BIC, separately for female and male students. For objective two, in order to compare the levels of BIC and its bivariate association with BMI, lifestyle, and mental well-being variables, chi-square test (χ^2^) assessed the overall differences in frequencies between levels of BIC, for females and males separately. Significance level was set at *p* < 0.005 in order to adjust for multiple comparisons.

For objective three, multifactorial linear regression analysis examined the association across BIC (dependent variable), and BMI, lifestyle factors (PA, nutrition, smoking) and mental well-being variables (QoL, perceived stress, perceived health, depressive symptoms), and body image perception (independent variables) for men and women separately. Results of the multifactorial linear regression analyses were expressed as adjusted R^2^ and standardized coefficient β, with *p* set at < 0.05.

## 3. Results

### 3.1 Prevalence and Associations of Three Levels of Body Image Concerns by Gender

Approximately 52.5% (n=1663) of participants were females, and the mean age was 19.3 (SD 1.6) and 18.6 (SD 1.2) years respectively for males and females. Tables 1 and 2 depict that 8.3% (males) and 15.8% (females) had moderate/marked BIC, while 17.3% and 24.2% of males and females respectively had mild BIC. Based on self-reported weight, 24.6% (males) and 32.5% (females) of the students were overweight.

**Table 1:** Body image concern and associations with other variables among male students in Egypt*

Males					
	**No BIC**	**Mild BIC**	**Moderate/Marked BIC**	***P*^a^**	**Total**
	1083 (74.4)	252 (17.3)	121 (8.3)		
	
**Variable**	n (%)	n (%)	n (%)		n (%)

BMI				**<0.001**	
*Underweight*	78 (7.4)	11 (4.5)	5 (4.3)		94 (6.6)
*Normal*	814 (76.9)	120 (49.6)	40 (34.5)		974 (68.7)
*Overweight*	167 (15.8)	111 (45.9)	71 (61.2)		349 (24.6)

Body image perception				**<0.001**	
*Just right*	718 (67.9)	102 (41.6)	32 (27.6)		852 (60.0)
*Too thin*	197 (18.6)	33 (13.5)	8 (6.9)		238 (16.8)
*Too fat*	143 (13.5)	110 (44.9)	76 (65.5)		329 (23.2)

**Lifestyle variables**					

Physical Activity				0.376	
*Not achieve PA guidelines*	431 (54.8)	94 (52.8)	39 (47.0)		564 (53.9)
*Achieve PA guidelines*	355 (45.2)	84 (47.2)	44 (53.0)		483 (46.1)
Nutrition (High calorie diet score)				0.294	
*Low*	482 (45.9)	121 (49.6)	58 (51.3)		661 (47.0)
*Medium*	288 (27.5)	61 (25.0)	21 (18.6)		370 (26.3)
*High*	279 (26.6)	62 (25.4)	34 (30.1)		375 (26.7)
Smoking				**0.016**	
*Non / occasionally*	1002 (93.2)	238 (96.7)	106 (89.1)		1346 (93.5)
*Daily*	73 (6.8)	8 (3.3)	13 (10.9)		94 (6.5)

**Mental well-being variables**					

Quality of Life				0.232	
*Lower*	605 (56.5)	147 (58.8)	76 (64.4)		828 (57.5)
*Higher*	466 (43.5)	103 (41.2)	42 (35.6)		611 (42.5)
Finance (stressor)				**<0.001**	
*Lower stress level*	921 (86.2)	198 (78.9)	83 (68.6)		1202 (83.4)
*Higher stress level*	148 (13.8)	53 (21.1)	38 (31.4)		239 (16.6)
Perceived stress				0.099	
*≤ Median*	738 (68.1)	157 (64.1)	76 (63.9)		971 (68.5)
*> Median*	315 (29.9)	88 (35.9)	43 (36.1)		446 (31.5)
Self-rated Health				0.060	
*Higher health*	265 (24.7)	49 (19.9)	20 (16.7)		334 (23.2)
*Lower health*	810 (75.3)	197 (80.1)	100 (83.3)		1107 (77.8)
Depressive symptoms score				**<0.001**	
*Low*	914 (88.1)	180 (76.3)	74 (67.3)		1168 (84.5)
*High*	123 (11.9)	56 (23.7)	36 (32.7)		215 (15.5)

* Bivariate associations;

a χ^2^-test to compare levels of BIC; all cell percentages are column percentages; bolded p values indicate statistically significant differences after adjusting the significance level to *p* <0.005 due to multiple comparisons.

**Table 2:** Body image concern and associations with other variables among female students in Egypt*

Females					
	**No BIC**	**Mild BIC**	**Moderate/Marked BIC**	***P*^a^**	**Total**
	966 (60.0)	390 (24.2)	254 (15.8)		
	
**Variable**	n (%)	n (%)	n (%)		n (%)

BMI				**<0.001**	
*Underweight*	66 (7.0)	14 (3.7)	4 (1.6)		84 (5.4)
*Normal*	696 (74.1)	215 (57.2)	60 (24.2)		971 (62.1)
*Overweight*	178 (18.9)	147 (39.1)	184 (74.2)		509 (32.5)

Body image perception				**<0.001**	
*Just right*	657 (70.3)	177 (46.9)	38 (15.2)		872 (55.9)
*Too thin*	122 (13.1)	34 (9.0)	8 (3.2)		164 (10.5)
*Too fat*	155 (16.6)	166 (44.0)	204 (81.6)		525 (33.6)

**Lifestyle variables**					

Physical Activity				0.484	
*Not achieve PA guidelines*	264 (40.6)	111 (41.0)	75 (45.7)		450 (41.5)
*Achieve Pa guidelines*	386 (59.4)	160 (59.0)	89 (54.3)		635 (58.5)
Nutrition: (High calorie diet score)				0.161	
*Low*	340 (36.3)	151 (39.5)	107 (44.0)		598 (38.3)
*Medium*	257 (27.5)	110 (28.8)	59 (24.3)		426 (27.3)
*High*	339 (36.2)	121 (31.7)	77 (31.7)		537 (34.4)
Smoking				NC	
*Non / occasionally*	912 (100)	362 (100)	248 (99.4)		1522 (99.9)
*Daily*	0 (0)	0 (0)	1 (0.4)		1 (0.1)

**Mental well-being variables**					

Quality of Life				**0.007**	
*Lower*	600 (62.4)	255 (66.2)	185 (72.8)		1040 (65.0)
*Higher*	361 (37.6)	130 (33.8)	69 (27.2)		560 (35.0)
Finance (stressor)				0.140	
*Lower stress level*	792 (83.0)	327 (85.2)	201 (79.1)		1320 (82.9)
*Higher stress level*	162 (17.0)	57 (14.8)	53 (20.9)		272 (17.1)
Perceived stress				**<0.001**	
*≤ Median*	572 (60.6)	196 (51.6)	111 (44.4)		879 (55.8)
*> Median*	372 (39.4)	184 (48.4)	139 (55.6)		695 (44.2)
Self-rated Health				0.329	
*Higher health*	144 (15.0)	47 (12.2)	40 (15.7)		231 (14.5)
*Lower health*	814 (85.0)	339 (87.8)	214 (84.3)		1367 (85.5)
Depressive symptoms score				**<0.001**	
*Low*	763 (82.8)	282 (75.6)	159 (64.9)		1204 (78.2)
*High*	159 (17.2)	91 (24.4)	86 (35.1)		336 (21.8)

* Bivariate associations;

a χ^2^-test to compare levels of BIC; all cell percentages are column percentages; bolded *p* values indicate statistically significant differences after adjusting the significance level to *p* <0.005 due to multiple comparisons; NC: not computed.

Tables 1 and 2 also show that among students with moderate/marked BIC, 32.7% (male) and 35.1% (female) had high depressive symptoms score (*p* <0.001 for both genders), 61.2% (male) and 74.2% (female) were overweight (*p* <0.001 for both genders), and 65.5% (male) and 81.6% (female) perceived their body image as too fat (*p* <0.001 for both genders). Only among male students, 31.4% with moderate/marked BIC had higher finance related stress level (*p* <0.001), and 10.9% smoked daily (*p* = 0.016). Likewise, only among female students, 72.8% with moderate/marked BIC reported lower QoL (*p* = 0.007), and 55.6% had higher perceived stress (*p* <0.001). As only one female in the sample reported daily smoking, this variable was excluded from subsequent analysis among females.

### 3.2 Correlates of Body Image Concern among Male Students (Multifactorial Analysis)

Table 3 shows that for male students, the model explained 27% of the BIC variance (adjusted R^2^ = 0.269). BMI, body image perception as being too fat, and depressive symptoms were positively associated with BIC, while self-rated health was negatively associated with BIC. The variables with the highest standardized β were BMI (0.29) and depressive symptoms (0.29). The remaining variables (PA, nutrition, smoking, QoL, finance-related stress, perceived stress score) were not associated with increasing BIC among males.

**Table 3:** Correlates of BIC among male students in Egypt

Variable	Standardized coefficient β	*P*
BMI	**0.29**	**<0.001**

Body image perception	**0.16**	**<0.001**

Lifestyle variables		

Physical activity	0.01	0.633
High calorie diet score	-0.04	0.180
Smoking	0.03	0.317

Mental well-being variables		

Quality of life	-0.02	0.512
Financial stress	0.02	0.488
Perceived stress score	0.02	0.542
Self-rated health	**-0.12**	**<0.001**
Depressive symptoms score	**0.29**	**<0.001**

Multifactorial linear regression, adjusted for age; Adjusted R^2^ = 0.269; Standardized coefficients β in bold indicate statistical significance.

### 3.3 Correlates of Body Image Concern among Female Students (Multifactorial Analysis)

Table 4 shows that for females, the model explained 41% of the BIC variance (adjusted R^2^ = 0.408). BMI, body image perception as too fat and depressive symptoms were significantly positively associated with BIC, while self-rated health was negatively associated. The variables with the highest standardized β were BMI (0.31) and body image perception (0.30). The remaining variables (PA, nutrition, QoL, finance related stress, and perceived stress) were not significantly associated with rising BIC among females.

**Table 4:** Correlates of BIC among female students in Egypt

Variable	Standardized coefficient β	*P*
BMI	**0.31**	**<0.001**

Body image perception	**0.30**	**<0.001**

Lifestyle variables		

Physical activity	-0.05	0.051
High calorie diet score	-0.01	0.651

Mental well-being variables		

Quality of life	0.01	0.971
Financial stress	-0.04	0.129
Perceived stress score	0.03	0.275
Self-rated health	**-0.07**	**0.012**
Depressive symptoms score	**0.29**	**<0.001**

Multifactorial linear regression, adjusted for age; Adjusted R^2^ = 0.408; Standardized coefficients β in bold indicate significant differences.

## 4. Discussion

Body image is an important aspect of self-representation and self-evaluation throughout life, and a concern for both genders (Cash & Deagle, 1997; Grossbard, Neighbors, & Larimer, 2011). Sparse information exists on BIC among young adults in the EMR, despite evidence in the Arabic culture of growing preference for thinness which is becoming more desired than the plump body image of the past (Kabir, Zafar, & Waslien, 2013). With social stresses linked to the acceptability and approval of idealized physical appearance among university students, the current study bridges the gap in knowledge to describe the prevalence of BIC among male and female students, assess the bivariate associations of BIC with a range of variables, scrutinize the correlates of BIC for males and females, and examine whether these BIC correlates were different by gender. These features attach great importance to the findings of the current study, as it contributes to the evidence base research necessary for screening initiatives, prevention programmes and intervention strategies for these young adults who are foreseen to be tomorrow’s leaders.

### 4.1 Prevalence and Associations of Three levels of Body Image Concerns by Gender (Bivariate Associations)

As for objectives one and two of the study, we described the prevalence of BIC by gender and its bivariate relationships with BMI, perceived body perception, lifestyle characteristics and mental well-being variables. Across our undergraduate sample, about 25.6% of males and 40% of females had some level (mild, moderate or marked) of BIC. It is not easy to be able to compare our findings directly with findings of studies undertaken elsewhere due to geographical and methodological limitations in the published literature. First, few BIC studies were undertaken in the EMR so that comparisons are relevant, given that body image and BIC are influenced by the prevailing culture, predominant religious beliefs of the country/region where a study is undertaken (El Ansari et al., 2010a), and the general social context and norms (Eapen, Mabrouk, & Bin-Othman, 2006). Secondly, undertaking comparisons was challenging due to methodological issues emanating from the different research tools/inventories employed by different studies to measure BIC (or body image dissatisfaction). For instance, in Iran, Akbarbegloo et al. (2010) employed the Body Image Concern Inventory which consists of 19 items (Littleton, Axsom, & Pury, 2005), whereas we employed the Body Shape Questionnaire that consists of fewer items (*8 items*) (Cooper et al., 1987; Evans & Dolan, 1993). However, the shortened 8 item version is sufficiently well validated to be used and comparable to other body shape/image questionnaires (Evans & Dolan, 1993).

Nevertheless, our findings compared favorably with Iran (65% of respondents reported body dissatisfaction) (Garousi & Nejad, 2014); and with UAE studies (Thomas et al., 2010; Schulte & Thomas, 2013) where 74.8% and 73% of the surveyed students respectively were dissatisfied with their current estimated body image, or felt body dissatisfaction as a concern. Such BIC is important as thin body preoccupation and social factors were critical in the development of abnormal eating attitudes (Eapen et al., 2006). In addition to the methodological challenges noted above, other factors might also contribute to explain our relatively lower levels of BIC. Firstly, our sample was from Assiut University in south Egypt which is relatively more conservative when compared with the liberal North part of Egypt. Secondly, in a country of predominantly the Muslim faith, females at university in south Egypt would probably wear long Islamic-style loose clothing (do not reveal body contours/silhouette) and a veil (Hijab) covering the head and upper body, and could reach down below the waist/hips. These features (conservatism and Islamic style clothing) might collectively render females in our sample to be relatively less concerned about their body image than females living in a more ’physically liberal’ region or country.

As for gender differences, we found that a higher percentage of females reported body image concerns (25.6% males and 40% females had some level of BIC; and about double the proportion of females compared to males had moderate/marked BIC). This is not surprising and we are in agreement with the USA, where college females were associated with greater body dissatisfaction (Blow & Cooper, 2014); and with Lebanon, where males were less worried about their body shape (Yahia et al., 2011). Similarly, UAE undergraduates reported body dissatisfaction as a concern, and local eating disorders prevention strategies needed to address the needs of both genders (Schulte & Thomas, 2013). Others have noted that women might have a more “negative” attitude towards their bodies, and the desire to be thin is a critical factor in women’s outlook toward their body image perception (Kiefer, Leitner, Bauer, & Rieder, 2000). Certainly, men too have become increasingly dissatisfied with their physical appearance; and men’s concerns include weight and muscularity (Frederick et al., 2007). Males have less BIC compared to females, perhaps because men find a greater range of ideal body image more socially accepted (e.g., being muscular) (Grossbard et al., 2011), whereas females might have a narrower variety of ideal body image (e.g., being thin/slim) (El Ansari et al., 2010).

In terms of BIC and its bivariate relationships with BMI, body image perception and general health, our findings support the literature. In our sample moderate/marked BIC was associated with an image perception of being too fat and also with overweight BMI and lower self rated health. We agree with other researchers, body image satisfaction was significantly associated with image perception and general health status (Goswami, Sachdeva, & Sachdeva, 2012). Additionally, Goswami et al. (2012), noted that students with BMI <18.5 kg/m^2^ had significantly higher prevalence of body image satisfaction, while overweight students had a significantly higher prevalence of dissatisfaction (Goswami et al., 2012).

As for smoking and BIC in our sample, only one female (<1%) reported smoking daily, compared to 6.5% males, a gender distribution that is supported by other EMR studies (Tessier, Nejjari, & Bennani-Othmani, 1999). Although smoking among university students in Egypt seems to be slightly less common compared to western countries (El Ansari et al., 2012a), nevertheless, the trend seems to be the same: daily smokers are more likely to have BIC compared to non/occasional smokers (Kendzor et al., 2009). Situational challenges to body image influence smoking motivation partially through increased negative affect (Lopez Khoury, Litvin, & Brandon, 2009), and despite the negative health effects, many young people smoke for weight and body image reasons (Napolitano, Lloyd-Richardson, Fava, & Marcus, 2011).

As regards to depressive symptoms and BIC, only a small proportion of our students with high depressive symptoms had no BIC (11.9% males, 17.2% females) (Tables 1, 2). This is important for educators and health policy makers, as depressive symptoms are associated with BIC, and also with body and weight dissatisfaction (Schulte & Thomas, 2013). We are in agreement with others e.g., in Trinidad and Tobago, body dissatisfaction was associated with higher risk for depression and lower self-esteem (Nichols, Dookeran, Ragbir, & Dalrymple, 2009); in the USA, greater body dissatisfaction was associated with greater depressive symptoms (Blow & Cooper 2014); and in the UAE, body dissatisfaction was a concern and future research needed to consider potential depressive co-morbidity (Schulte & Thomas, 2013).

### 4.2 Correlates of Body Image Concerns among Female and Male Students (multifactorial analysis)

For objective three, the correlates of BIC in both genders did not exhibit striking differences. Among both genders, the same four variables (BMI, depressive symptoms score, body image perception and self-rated health) were significantly associated with BIC. Likewise, in male and female students, both BMI and depressive symptoms exhibited stronger associations with BIC than self rated health. However, body image perception had stronger associations with BIC (about double) in females than in males. Self-rated health had the least effect on BIC.

It is important to note that the variables associated with BIC (e.g., BMI, depressive symptoms, body image perception and self-rated health) in the current study, do not operate in isolation and need not be viewed in a sequestered (silo) manner. The relationships are complex. For instance perceived body image and a desire to be thinner were strongly related to body image dissatisfaction (Kabir et al., 2013); depression is associated with body weight dissatisfaction and poor body image (Santos, Richards, & Bleckley, 2007); body image perceptions entirely mediated the association between higher BMI levels and higher depressive symptoms (Eidsdottir, Kristjansson, Sigfusdottir, Garber, & Allegrante, 2013); and body image dissatisfaction mediated the relationship between BMI and quality of life in both genders (Wilson, Latner, & Hayashi, 2013). These examples demonstrate the multiple patterns, complex configurations and interlacing nature of the variables and concepts associated with BIC.

Although the literature suggested several factors as potentially being associated with BIC like nutrition, PA, smoking, QoL, and perceived stress, this was not totally supported by our current data. Whilst in our bivariate analyses, smoking and financial stressors (males) and quality of life (females) exhibited significant associations with BIC, these effects were lost in the multifactorial analyses suggesting that these observed effects were moderated through other variables.

Limitations of the study

This current study has various limitations. As a cross-sectional survey, the directions of effects cannot be ascertained (findings are associations not causations). We surveyed eleven faculties in a single university in Egypt, and although it is not uncommon practice in student surveys, it remains a convenience sample (El Ansari et al., 2011a; Lee & Loke, 2005). The comparison of male and female student BIC was measured by the Body Shape Questionnaire (Cooper et al., 1987) comprising of questions that were originally developed based on females’ BIC. Therefore it might not be as adequate regarding measuring males’ BICs. Some concerns that are more typical for males might be lacking from the questionnaire, which could then lead to underestimating males’ actual BIC. Self-reported data might include recall bias, sociability and social desirability. Students who participated might be those with better health during the data collection day/s. Therefore, the sample’s BIC level might underestimate the BIC across larger student samples. As the study was a general health survey, and in order to decrease respondent burden, we assessed some variables by single item measures as the survey was conducted within a short time in classes, rendering the use of in depth measures for each health factor unfeasible. Future research should try to address these limitations.

## 5. Conclusion

We conclude that BIC may exist to an extent among male and female undergraduate students at Assiut University in Egypt, but the prevalence is significantly higher among females. The variables associated with BIC did not differ substantially between male and female students. BMI, depressive symptoms, body image perception as being too fat, and self-rated health were associated with BIC for both genders, a point that needs to be considered when developing health promotion programs that address mental well-being and BIC. In addition, males’ BIC still requires more attention, and also necessitates the development/refinement of methods to specifically assess male BIC.
